# Profiling of Taxoid Compounds in Plant Cell Cultures of Different Species of Yew (*Taxus* spp.)

**DOI:** 10.3390/molecules28052178

**Published:** 2023-02-26

**Authors:** Dmitry V. Kochkin, Elena V. Demidova, Elena B. Globa, Alexander M. Nosov

**Affiliations:** 1 Timiryazev Institute of Plant Physiology, Russian Academy of Sciences, Botanicheskaya Str. 35, 127276 Moscow, Russia; 2 Faculty of Biology, Lomonosov Moscow State University, Leninskie Gory 1-12, 119234 Moscow, Russia

**Keywords:** plant cell culture, *Taxus* spp., taxoids, 14-hydroxylated taxoids

## Abstract

Plant cell cultures of various yew species are a profitable source of taxoids (taxane diterpenoids) with antitumor activity. So far, despite intensive studies, the principles of the formation of different groups of taxoids in cultured in vitro plant cells have not been fully revealed. In this study, the qualitative composition of taxoids of different structural groups was assessed in callus and suspension cell cultures of three yew species (*Taxus baccata, T. canadensis*, and *T. wallichiana*) and two *T. × media* hybrids. For the first time, 14-hydroxylated taxoids were isolated from the biomass of the suspension culture of *T. baccata* cells, and their structures were identified by high-resolution mass spectrometry and NMR spectroscopy as 7β-hydroxy-taxuyunnanin C, sinenxane C, taxuyunnanine C, 2α,5α,9α,10β,14β-pentaacetoxy-4(20), 11-taxadiene, and yunnanxane. UPLC–ESI-MS screening of taxoids was performed in more than 20 callus and suspension cell lines originating from different explants and grown in over 20 formulations of nutrient media. Regardless of the species, cell line origin, and conditions, most of the investigated cell cultures retained the ability to form taxane diterpenoids. Nonpolar 14-hydroxylated taxoids (in the form of polyesters) were predominant under in vitro culture conditions in all cell lines. These results, together with the literature data, suggest that dedifferentiated cell cultures of various yew species retain the ability to synthesize taxoids, but predominantly of the 14-OH taxoid group compared to the 13-OH taxoids found in plants.

## 1. Introduction

Diterpenoids of the taxane group (taxoids) are specific for plants of the genus *Taxus* (yew, Taxaceae). More than 300 individual taxoid compounds have been isolated from different yew species [1,2]. These compounds can be divided into several classes based on the structure of the taxane skeleton and the nature and/or arrangement of the functional groups [1,2]. Three to six structural classes of taxoids are usually distinguished; however, the representatives of three groups are the most common among *Taxus* spp.: 13-hydroxylated taxoids of the baccatin III-type (baccatin III, paclitaxel, etc.), 14-hydroxylated taxoids of the taiwanxan-type (taxuyunnanin C, yunnanxane, etc.), and 11(15→1)-*abeo*-taxoids [1,2,3,4]. Paclitaxel (commercial synonym Taxol^®^) is a pharmaceutically important chemical that is widely used in cancer therapy [1].

The mechanism of taxol action is unique as it inhibits microtubule depolymerization. Taxol penetrates into cells and disrupts cytoskeleton functions, which causes the suppression or malfunctioning of various processes in eukaryotic cells. These include inhibition of cell proliferation and intracellular mobility, alterations of membrane structure and function, disruption of intracellular transport, compartmentation, signaling, etc. [5]. Taxol is highly effective in treating breast, ovarian, and lung cancer, which are the world’s most common cancer types [6]. Recently, however, the use of taxol has been hampered by a major concern—the emergence and development of tumor cells’ resistance to first-generation chemotherapeutic taxane drugs (13-hydroxylated taxoids (taxol) and its semisynthetic derivatives docetaxel, cabazitaxel, etc.) [7]. A pharmacological study of taxoids of other structural types showed that some taxoids, despite being structurally different from taxol, exhibited cytotoxic activity comparable to that of taxol derivatives in relation to certain tumor cell lines [8]. However, it was found that these “unusual” from the perspective of classical taxane diterpenoids chemotherapy agents are effective against tumor cell lines with a multidrug resistance phenotype [8,9]. Several taxoids, including 14-hydroxylated ones, can suppress the resistance of tumor cells to cytotoxic compounds by changing the work of transporters and the plasma membrane (in particular, ABC-transporters) [10]. Taxoids can inhibit epidermal growth factor receptor tyrosine kinase [11] and act as immunomodulators to activate the antitumor properties of effector cells [12]. Consequently, not only taxol but other taxoids of different structural types can be used in cancer chemotherapy as independent drugs or components of complex treatments. In addition to antitumor action, different types of taxoids demonstrate other biological activities, including antidiabetic (by inhibiting alpha-glucosidases and insulin resistance caused by inflammation, and disrupting the lipoxygenase cascade), anti-inflammatory (by impairing migration of leukocytes and development of a granuloma in the site of inflammation), analgesic, antipyretic, anticonvulsant (probably by modulating GABA_A_ receptors), inhibition the formation of superoxide anion radical in neutrophils (by impairing phosphorylation and intracellular transport of proteins-subunits of NADPH oxidase of the plasma membrane), antimicrobial, fungicidal, antileishmanial and several others [13,14,15,16,17,18,19,20]. Based on the information presented, it is important to perform a detailed phytochemical study of not only the baccatin III-group taxoids (paclitaxel and its derivatives), but also other taxane diterpenoids.

The specific biology of yew species, including endemism, slow growth, difficulties in reproduction, and the slow and unstable accumulation of paclitaxel and other taxoids (0.001–0.03% of the dry weight) in wild plants, significantly limit the industrial production of taxoids from natural plant sources [1,2,3,4].

Plant cell culture can serve as an alternative source of taxoids. There is a pool of publications describing cell cultures of various yew species and their ability to produce taxoids [21]. However, many authors noted either the absence or only trace amounts of taxoids in the cell cultures of *Taxus* spp. [22]. These conclusions were driven primarily by the analysis of 13-hydroxylated taxoids (mostly paclitaxel, baccatin III, and some others) in cell cultures [21,22]. Many 13-OH taxoids are polar compounds, which determines the strategy of their chemical analysis using modern HPLC/UPLC systems with reversed-phase adsorbents [23,24]. However, taxane diterpenoids are very diverse in both structure and physicochemical characteristics. Representatives of several taxoid classes, for example, 14-hydroxylated taxoids, are hydrophobic compounds with a longer elution time in reversed-phase HPLC compared to 13-OH taxoids [23,24]. As a result, these compounds may remain unrevealed during the chemical analysis of cell cultures of *Taxus* spp. which is usually focused primarily on 13-OH taxoids. The abovementioned considerations suggest that the formation of taxoids of various structural classes in cultured yew cells is relatively underexplored and requires further investigation. 

Furthermore, from a scientific viewpoint, isolated plant cells cultured in vitro are not analogous to the cells of whole plants [21]. As a result, many fundamentally important processes, including secondary metabolism, in cell cultures are different from those in plants [21,25]. Several studies demonstrated that the profile of secondary metabolites in plant cell cultures could be altered compared to their donor plants, which is often reflected in the promotion or suppression of the production of certain metabolite groups [21,25]. Meanwhile, there are very few publications related to the composition of structurally different taxoid groups in the in vitro cell cultures of different *Taxus* species [26,27,28,29]. 

In this work, we present for the first time a detailed UPLC–ESI-MS analysis of the structural diversity of taxoids in different lines (by explant origin) of callus and suspension cell cultures of three yew species (*Taxus baccata* L., *T. canadensis* Marshall, and *T. wallichiana* Zucc.) and two *T.* × *media* Rehder hybrids (*T.* × *media* cv. Aureovariegata and *T.* × *media* cv. Dovastaniana), grown in different nutrient media. The cell lines have been cultured in vitro for over 10 years, except for the cell culture of *T. wallichiana,* which is 5-years-old.

## 2. Results

### 2.1. Growth Characteristics of Taxus *spp*. Cell Cultures 

#### 2.1.1. Callus Cell Cultures

In this study, we used callus cell cultures of three yew species (*Taxus baccata*, *T. canadensis*, and *T. wallichiana*) and two *T.* × *media* hybrids: *T. × media* cv. Aureovariegata and *T. × media* cv. Dovastaniana. All callus and suspension cell cultures, except for *T. wallichiana*, were maintained by periodic subcultures for over 10 years and are represented by several (in some species, over 20) cell lines that originated from different explants. The *T. wallichiana* cell culture was maintained in an actively growing state for 5 years and is represented by a single line. The cultures were grown in more than 20 different media; the main differences in the media composition were growth regulators and the presence of antioxidants (polyvinylpyrrolidone) or adsorbents (activated charcoal). A complete list of the cell lines used and cultivation conditions is given in Table 1.

Table 1 presents an increase in the fresh weight of callus cell lines measured on the 56th day of cultivation. For the majority of cultures, the increase in cell biomass ranged from 1.5 to 5.0, which is consistent with the literature [30]. For three cell lines of *T. baccata* and two cell lines of *T. × media*, relatively high values of the growth index (7.5–9.1) were recorded, which significantly exceeded those reported in the literature [30]. No correlation was observed between fresh weight accumulation and species, explant, or medium composition (Table 1). However, cell cultures originating from the 40-year-old tree in the botanical garden of the Moscow State University (lines Tb-msu) demonstrated very slow growth. Normally, growing cell cultures of this origin could be developed when the original callus line was cultured in liquid medium to form a cell suspension, followed by placing the cells back on solid medium (the “callus-suspension-callus” cycle). Callus cultures produced through this scheme demonstrated better growth than the original callus lines. In general, cell cultures growing in the presence of activated charcoal showed relatively high growth indices (from 3 to over 7.5).

#### 2.1.2. Suspension Cell Cultures

**Figure 1:**
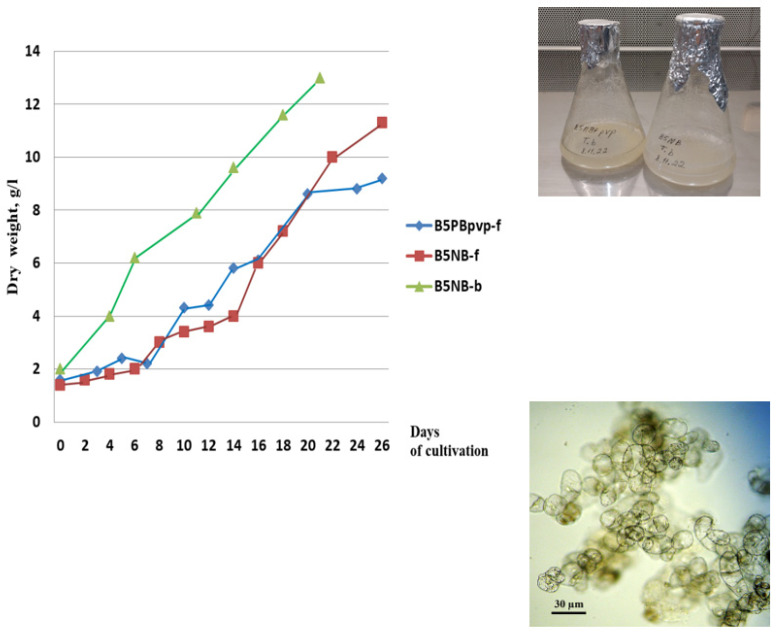
Left—Growth curves of the suspension cell culture of *Taxus baccata* lines **Tb-msu/**B5-PB-*pv*p (**B5PBpvp-f** in the legend) and **Tb-msu/**B5-NB (**B5NB-f**) during cultivation in flasks and line **Tb-msu/**B5-NB during cultivation in 20-L bioreactor (**B5NB-b**) under semi-continuous regime (the third cycle of cultivation). Right—photograph of cell suspension cultures in 250-mL flasks and microphotograph of cells of **Tb-msu/**B5-NB line.

Suspension cell cultures developed in this study had different but generally high growth characteristics. The maximum accumulation of dry biomass *M*_max_ for all studied cell lines was within 7–16 g/L; growth index *I* ranged from 4 to 10; specific growth rate μ was 0.10–0.22 day^–1^; economic coefficient *Y* was 0.1–0.3; and biomass productivity *P* was 0.2–0.8 g/L per day. The highest growth parameters were recorded for suspension cell lines **TmA-msu**/B5-NB, **Tb-msu**/B5-NB, **Tb-msu**/B5-PB, and **Tb-msu**/B5-DK. Representative growth curves of cell lines **Tb-msu/**B5-PB-*pv*p and **Tb-msu/**B5-NB are shown in Figure 1. 

In order to accumulate a sufficient amount of biomass for the isolation of taxoids, suspension cell culture **Tb-msu**/B5-NB-ac (*Taxus baccata*) was cultured in a 20-L bubble-type bioreactor operated in a semi-continuous mode for four sequential growth cycles. The growth parameters of the cell culture improved gradually upon cell adaptation to bioreactor conditions (maximum biomass accumulation increased from 6.5 to 15 g/L, specific growth rate—from 0.12 to 0.20 day^−1^). Cell viability in all growth cycles was above 90%. A representative growth curve of the **Tb-msu/**B5-NB cell line during the third subculture cycle in the bioreactor is given in Figure 1. 

The main growth parameters of the suspension cell culture in the subculture cycle shown in Figure 1 are presented in Table 2.

### 2.2. Phytochemical Screening of the Cell Cultures of Taxus *spp*.

#### 2.2.1. Structural Identification of Taxoids in Cell Cultures

The first step in the phytochemical analysis was a detailed structural identification of toxoids present in the cell cultures. A UPLC–ESI-MS analysis of the taxoid composition was performed using biomass from callus cell cultures of *T. baccata* line **Tb-nbg**/R-NB-ac, grown on B5-PB-ac medium. This cell line was maintained by periodic subcultures for more than 10 years and showed the highest increase in fresh weight. Callus was taken to the lab for analysis on day 74 of the 42nd subcultivation. The positive ion detection mode (electrospray ionization) was selected to record chromatograms and mass spectra since it allows gathering the most information on the structure of taxane diterpenoids, as well as other natural compounds, during a single run due to molecule fragmentation in the ionization source [25,31].

**Figure 2:**
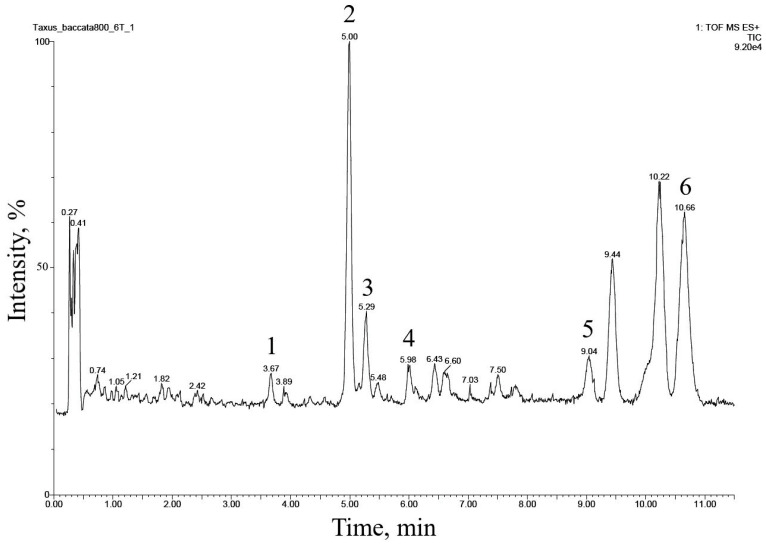
UPLC–ESI-MS chromatogram (total ion current, positive ion mode) of an alcohol extract from the biomass of *T. baccata* callus cell culture (**Tb-nbg** /R-NB-ac line grown in a B5-PB-ac medium, 74 days of 42nd subcultivation). The description of peaks **1**–**6** is given in Table 3.

The UPLC–ESI-MS chromatogram of the alcohol extract from this cell culture recorded in the total ion current mode (positive ions) is presented in Figure 2. Six peaks of compounds were found and eluted from the column within 3.5–11 min. The comparison of their MS spectra with the literature data suggested that they belong to the diterpenoids of the taxane group. These compounds were numbered **1** through **6** in order of increasing hydrophobicity, that is, increasing retention time on a reversed-phase chromatographic column.

The preliminary analysis of the MS spectra of the detected compounds indicated (Table 3) that all of them belong to neutral taxoids with molecules without nitrogen-containing functional groups. This conclusion was supported by the fact that ions with odd *m*/*z* values predominated in the MS spectra of all compounds. In addition, intense signals of adduct ions [M + NH_4_]^+^ and [M + Na]^+^ were observed, and there were almost no signals of protonated ions [M + H]^+^ (Table 3) [23,24,28].

The fragmentation of compounds **1**–**6** in the ionization source suggested that they all belong to the so-called “regular” taxoids containing a taxa-4(20),11-diene skeleton [1,23,24,28]. Based on the number of substituents in taxa-4(20),11-diene core fragment, compounds **1**–**6** could be divided into two structural subclasses: (1) compounds **1**, **2**, and **5**, derivatives containing five substituents (the presence of a pair of characteristic ions with *m*/*z* 281 and 263), and (2) compounds **3**, **4**, and **6** containing four substituents (the presence of a pair of characteristic ions with *m*/*z* 283 and 265) [1,23,24,28].

By their chemical nature, the substituents in the taxadiene skeleton of the identified compounds are hydroxyl groups esterified (in various combinations) with aliphatic acid residues [1,23,24,28]. The following acyl substituents were identified: acetic acid (identified in all compounds based on the presence of neutral losses of 77 (C_2_H_4_O_2_ + NH_3_), 60 (C_2_H_4_O_2_), and/or 42 Da (C_2_H_2_O) upon fragmentation of the [M+NH_4_] adduct ion in the ionization source), hydroxymethylbutanoic acid (for compound **3**, neutral loss of 118 Da (C_5_H_10_O_3_)), methylbutanoic acid (for compounds **5** and **6**, neutral loss of 119 Da (C_5_H_10_O_2_ + NH_3_)).

The described patterns of MS fragmentation (positive ion mode) suggested that taxoids **1**–**6** belonged to the structural group of taiwanxan (14-hydroxylated taxoids). 

**Figure 3:**
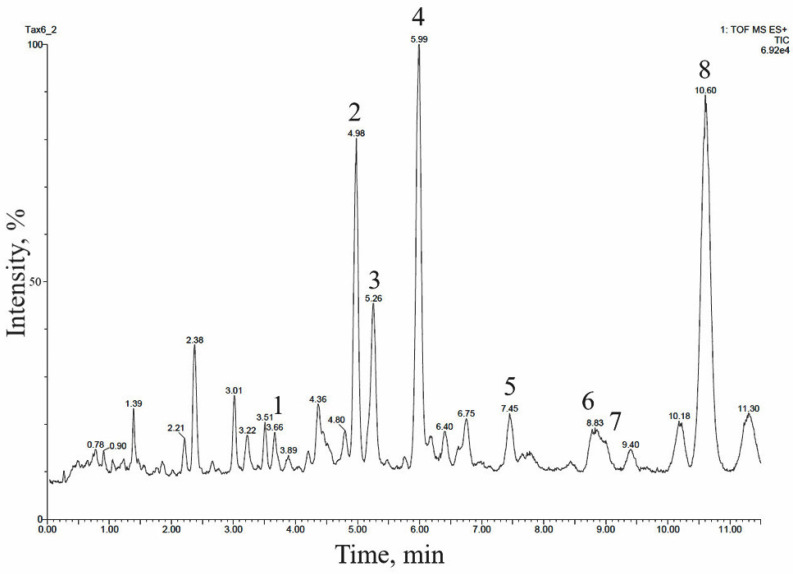
UPLC–ESI-MS chromatogram (total ion current, positive ion mode) of an alcohol extract from the biomass of *T. baccata* cell suspension culture (**Tb-msu**/B5-NB-Car line grown in a 20-L bioreactor). The description of peaks **1**–**8** is given in Table 4.

The 14-hydroxylated taxoids were also predominant in the extract of the cell biomass of a suspension culture of *T. baccata*, line **Tb-msu**/B5-NB, grown in a 20-L bioreactor, as confirmed by UPLC–ESI-MS (Figure 3 and Table 4). Many of these compounds were identical, in terms of relative chromatographic behavior and mass spectrometry data, to taxoids identified in *T. baccata* callus culture.

In order to verify the structural identification of the detected taxoids, we isolated major diterpenoids from 93 g of air-dried biomass from a suspension culture of *T. baccata* (**Tb-msu**/B5-NB) grown in a 20-L bioreactor. 

Five taxoids were present in the cell biomass in amounts sufficient for preparative isolation (analytical TLC results); they were identified by specific staining (pink-lilac color) upon the development of the TLC plate with an anisaldehyde–sulfuric acid reagent [1]. The detected compounds were designed in decreasing order of polarity (relative mobility (*R*_f_) in the ethyl acetate–hexane system (1:1, *v*/*v*)) as follows: **I** (*R*_f_ 0.3), **II** (*R*_f_ 0.5), **III** (*R*_f_ 0.7), **IV** (*R*_f_ 0.8), and **V** (*R*_f_ 0.9). Using conventional column chromatography and semipreparative TLC, these compounds were isolated in pure form with the following yields (% of dry cell mass): **I** 0.0006%, **II** 0.0030%, **III** 0.0050%, **IV** 0.0080%, and **V** 0.014%. The structures of the isolated glycosides were determined using high-resolution mass spectrometry and NMR spectroscopy.

**Figure 4:**
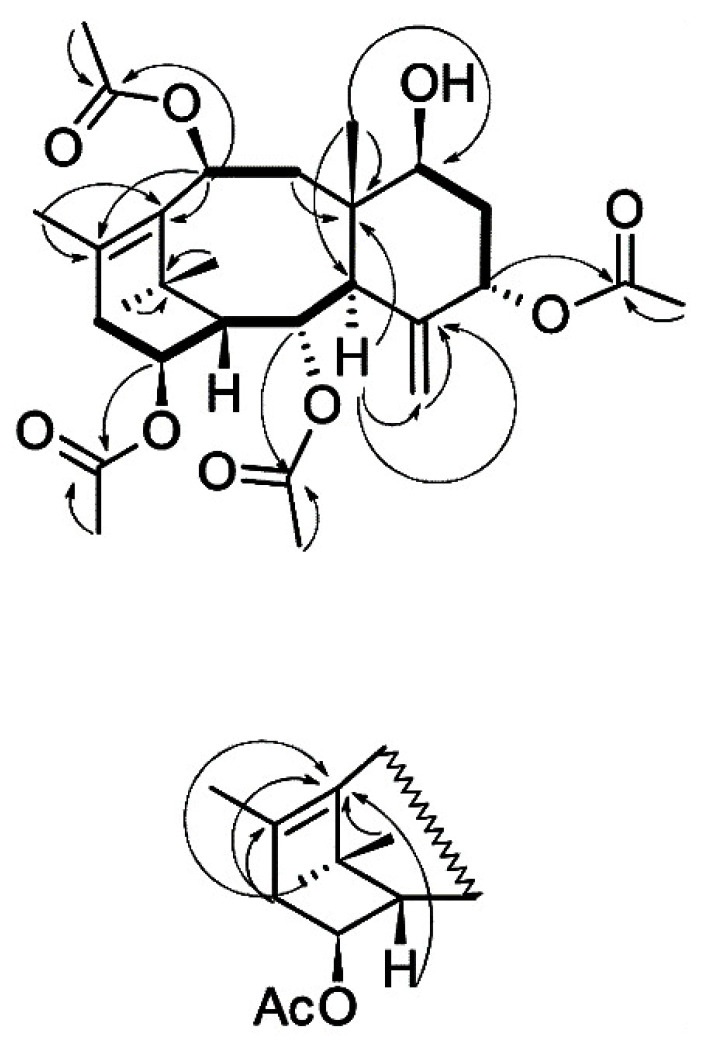
Structure and key ^1^H–^1^H COSY (bold lines) and ^1^H–^13^C HMBC (arrows) correlations of taxoid **I** isolated from *T. baccata* cell suspension culture.

The results of the interpretation of the ^13^C NMR spectrum of taxoid **I** suggest that the isolated compound has the skeleton of taxa-4(20),11-diene substituted with hydroxyl groups at positions C2, C5, C7, C10, and C14 (Supplementary Table S1). The hydroxyl groups at C2, C5, C10, and C14 are esterified with four acetic acid residues. The order of attachment of acyl fragments to the diterpene backbone was determined by interpreting the results of the ^1^H–^13^C HMBC experiment (Figure 4). The stereochemistry of substituents in the molecule of compound **I** was determined based on the analysis of the spin-spin coupling constants of the corresponding protons and their comparison with the published data (Supplementary Table S2) [27,28,29,32].

Thus, the interpretation of the ^1^H and ^13^C NMR spectra of the compound **I** and analysis of the literature data [27,28,29,32] suggest that this taxoid has the structure of 7β-hydroxy-2α,5α,10β,14β-tetraacetoxy-4(20),11-taxadiene (Figure 4) and corresponds to 7β-hydroxy-taxuyunnanine C, which was first isolated from *T. cuspidata* cell culture [32]. The described structure is also consistent with the results of high-resolution mass spectrometry of the isolated taxoid: the formula C_28_H_40_O_9_ is confirmed by the presence of a signal of the [M + Na]^+^ adduct ion at *m*/*z* 543.2572 in the spectra of positive ions of this compound (calculated value *m*/*z* 543.2565). 7β-Hydroxy-taxuyunnanine C can be classified as a rare and/or unusual taxoid: it is one of a few 14-hydroxylated taxoids having a hydroxyl group at the C7 position of 4(20),11-taxadiene; only two taxoids with a similar structure are currently known [1,2,3,4]. There is only one report on discovering this taxoid first isolated from *T. cuspidata* callus cell culture [32]. Thus, 7β-hydroxy-taxuyunnanine C was detected for the first time in *T. baccata* cell culture.

NMR spectroscopy and high-resolution mass spectrometry of compounds **II**–**V** were performed in a similar way and revealed that they have the structures of sinenxane C, taxuyunnanine C, 2α,5α,9α,10β,14β-pentaacetoxy-4(20),11-taxadiene, and yunnanxane, respectively (Supplementary Tables S1 and S2). These taxoids were also previously isolated from cell cultures of different yew species (*T. chinensis*, *T. chinensis var. mairei*, *T. × media*, *T. wallichiana*, and *T. cuspidata*) [23,24,25,26,27,28,29,33,34]. However, these 14-hydroxylated taxoids were found for the first time in *T. baccata* cells cultured in vitro.

Thus, the preparative isolation and structural study of individual taxoids confirm the results of their identification performed using UPLC–ESI-MS. 14-hydroxylated taxoids predominated in callus and suspension cultures of *T. baccata* cells.

#### 2.2.2. Screening of Taxoids in the Cell Cultures of Various Yew Species, Provenance and Cultivation Conditions

In order to reveal any general pattern in the accumulation of taxoids of different structural groups among the cell cultures of *Taxus* spp., we performed the UPLC–ESI-MS phytochemical screening of taxoids from all available cell cultures (over 20 cell lines in total). These cell lines belonged to different yew species and were induced from different donor plants using different explants in different media. Identification of 14-OH taxoids was accomplished by comparing their retention times and mass spectra with standard samples of 14-OH taxoids isolated at the first step of this study. Sinenxane B and taxuyunnanine B were identified by comparing the results of mass spectrometry with the literature [23,24]. Commercial standard samples of Baccatin III, 10-deacetyl-7-xylosyl taxol, cephalomannine, paclitaxel, and taxusin were used to identify 13-hydroxylated taxoids.

The results of screening the biomass of callus cell cultures are presented in Table 5. 

As a result, 13-hydroxylated taxoids (paclitaxel, baccatin III, etc.) were not detected in the cell samples of any callus cell cultures of *Taxus* spp., while 14-hydroxylated taxoids were found in the biomass of almost all studied callus lines. Only four out of the 45 samples tested did not have taxoids.

The screening results emphasized the role of the genotype (the type of plant used for culture induction) in the formation of taxoids in cell cultures. Among the studied callus cultures, *T. × media* cv. Dovastaniana showed the lowest number of toxoids, and their accumulation in the cell biomass was unstable: only 2 out of 4 biomass samples from this cell culture line contain 14-OH taxoids.

Similar results were obtained for suspension cultures of *Taxus* spp. grown in flasks (Table 6): 13-OH taxoids were absent in all samples, while 14-OH taxoids were found in most samples. 

At the same time, taxoids were not detected in any of the samples of suspension cultures of *T. × media* cells but were present, with one exception, in all analyzed samples of cell cultures of other species of *Taxus* spp. This confirmed the earlier conclusion about the low capability of *T. × media* cell cultures to form taxoids.

The qualitative composition of 14-OH taxoids in the biomass of *Taxus* spp. may somewhat vary depending on growing conditions. For example, out of the 8 taxoids found in the biomass of the suspension culture of *T. baccata* cells (**Tb-msu**/B5-NB) grown in a bioreactor, only sinenxane C is stably present in the cell culture grown in flasks.

#### 2.2.3. Screening of Taxoids Released to Cultivation Medium

Paclitaxel and some other 13-OH taxoids can be secreted into the apoplast and the growth medium during the in vitro growth of yew cells [35]. Therefore, we performed the UPLC–ESI-MS screening of culture media of callus and suspension cell cultures studied in this work.

As a result, 13-hydroxylated taxoids were not detected in any culture media samples (Supplementary Tables S4 and S5), while 14-OH were present in most media of the studied callus and suspension cell cultures. 14-OH taxoids were detected in 21 of 28 test samples of callus cultures and in 6 out of 8 samples of suspension cultures (Supplementary Tables S3 and S4). Taxoids were often absent in the culture media of *T. × media* cells: they were not detected in 4 out of 8 test samples of callus cultures and in none of the suspension cultures.

## 3. Discussion

There are a number of publications on plant cell cultures of different *Taxus* species. The main goal of the majority of those studies is practical: to obtain well-growing cell cultures and develop strategies for enhanced production of taxoids, primarily of commercially valuable paclitaxel [21].

Some authors reported the development of plant cell cultures accumulating taxol in amounts comparable to its content in plants; however, in most cases, taxol was absent in the cell cultures or present in trace amounts [22]. In suspension cell culture, paclitaxel was first discovered in 1989 in the cell culture of *T. brevifolia* [36].

Several trends could be found while analyzing the available literature sources on yew cell cultures [22,30,35]:

1. In most publications, studies were performed on relatively "young” cell cultures, 1-2 years after induction, while the content of secondary compounds may change in cell cultures during long-term cultivation.
2. The majority of the studies, with rare exceptions, only focused on the analysis of a few industrially valuable 13-OH-hydroxylated compounds (paclitaxel, baccatin III); other groups of taxoids were not screened.
3. Most studies were performed using cell cultures of one yew species only, which did not allow generalizing on potential trends of taxoid formation in the cell cultures of different *Taxus* species.

In the present study, taxoids of various groups were screened in the plant cell cultures of different *Taxus* species, and long-term (at least 10 years) grown cell cultures were mostly used for analysis.

The results indicate that, regardless of the species, cell line, or cultivation conditions, most of the investigated cell cultures of *Taxus* spp. retain the ability to form taxane diterpenoids. However, in the in vitro cultured yew cells, the metabolism of taxoids was shifted towards the predominant formation of non-polar 14-hydroxylated derivatives (in the form of polyesters), while more hydrophilic and toxic 13-oxygenated (in particular, 13-hydroxylated) taxoids were predominant in the aerial parts of intact plants that were used as explants for culture induction [1,2,3,4,21,22,23,24]. A comparison of the obtained results with literature data [26,27,28,29,33,34] confirms that this is a general pattern and is observed in plant cell cultures of almost all yew species, in which 14-OH taxoids were analyzed.

The reasons for such a metabolic shift may lie in the unique physiology of a plant cell culture as a population of dedifferentiated proliferating cells [21]. Many processes in cells grown in vitro, including secondary metabolism, differ significantly from those in whole plants due to the absence of organismic control, a different signaling system, and altered compartmentation [21]. The formation of 14-OH taxoids in plant cell cultures may be due to their lower toxicity for proliferating cells compared to 13-OH derivatives. For example, paclitaxel and some of its homologues disrupt the functioning of the cytoskeleton, which is lethal for most eukaryotic cells [37].

The pattern of taxoid accumulation described in the present work is unique compared to cell cultures of most other plant taxa, where the secondary metabolism upon cell dedifferentiation is usually shifted towards increased formation of more polar/hydrophilic compounds [21,25]. This might be explained by comparing the results of this study with phytochemical investigations of yew plants. *Taxus* species tend to accumulate hydrophobic secondary metabolites [4]. For example, phenolic compounds in yews are mainly represented by biflavones, lignans, esters of catechins, etc. In *Taxus* spp., glycosylated secondary metabolites (except for cyanogenic glycosides, some phenolic minor derivatives, xylosides, and very rarely taxoid glucosides) are less common [4,38] than in angiosperms [39].

The results reported here have both fundamental and applied significance, since they demonstrate the importance of *Taxus* spp. cell cultures as renewable and environmentally friendly sources of 14-hydroxylated taxoids. Taxoids with 14-hydroxylated structures have a wide range of practical uses. For example, 14-OH taxoids synthesized by in vitro cultured yew cells decrease tumor cell resistance to cytotoxic compounds through disruption of plasma membrane ABC transporters by direct non-covalent binding to these proteins and/or modulation of MAP signaling. Therefore, 14-OH taxoids can be used as components of complex cancer chemotherapy programs [10,40]. Furthermore, 14-OH taxoids isolated from yew cell cultures can be used as intermediate compounds for chemical modifications and biotransformation to generate new taxane drugs [41,42]. 14-OH taxoids can also be used to treat other diseases besides cancer. It has been demonstrated that many natural 14-OH taxoids act as effective alpha-glucosidase inhibitors and are useful in the treatment of diabetes [17]. The 14-OH taxoids can act as nerve growth factor (NGF) mimetics, making them useful in the prevention of side effects associated with classical cytostatics (taxol, cisplatin, and vincristine) and Alzheimer’s disease [4,42]. Accordingly, yew cell cultures can be used as sources of natural compounds important to human physiology and medicine. This study adds to the literature’s already established fact that yew cell cultures in vitro are an excellent source of raw material for isolating unusual and rare (not typical for intact yew plants) taxoids with unique biological properties [8].

## 4. Materials and Methods

### 4.1. Plant Material

#### 4.1.1. Callus Cell Cultures 

Callus cell cultures of three yew species (*Taxus baccata, T. canadensis*, and *T. wallichiana*) and two *T. × media* hybrids (*T. × media* cv. Aureovariegata and *T. × media* cv. Dovastaniana) were used in the study. Callus cultures of *T. baccata* cells were obtained in 2007–2009 from two plants: a 40-year-old tree from the Botanical Garden of the Moscow State University, Moscow (MSU Botanical Garden; Tb-msu line) and an 800-year-old tree from the Nikitsky Botanical Garden, Crimea (Tb-800 line). Callus cultures of *T. canadensis* were obtained in 2008 from a 40-year-old plant from the Botanical Garden of Moscow State University (lines Tc-msu); callus cultures of *T. × media* were developed in 2007–2008 from 30-year-old plants from the Botanical Garden of Moscow State University (lines TmD-msu (cv. Dovastaniana) and TmA-msu (cv. Aureovariegata)). A callus culture of *T. wallichiana* was induced in 2016 from a 50-year-old tree from the Central Botanical Garden of the National Academy of Sciences of Belarus, Minsk (NASB Botanical Garden; line TW-Bel). Stem segments (a small section of the stem with 1–3 leaves) were used as explants. Each line was obtained from a single explant on a specific medium. For each line, the induction medium is designated as “Im” (initial medium). The conditions for culture induction were described earlier [43]. 

The developed cell lines were grown on media of different compositions, as described below.

Three mineral salt formulations were used: Gamborg’s medium (B5), Reinart’s medium (R), and White’s medium (W). All media contained vitamins as described by Gamborg (nicotinic acid 0.5 mg/L, pyridoxine 0.1 mg/L, thiamine chloride 0.1 mg/L, Serva, St. Louis, Missouri, USA), 3% sucrose (Merck, Germany), and 0.55% agar (Merck, Germany).

The following combinations of growth regulators were used: NB—1-NAA (2 mg/L) and BAP (0.3 mg/L); PB—picloram (1 mg/L) and BAP (0.3 mg/L); DK—2,4-D (1 mg/L) and kinetin (0.3 mg/L); all growth regulators were purchased from Serva. The media also differed by the presence of polyvinylpyrrolidone (PanReac AppliChem, molecular weight 4000, 1.0 g/L) or activated charcoal (Fluka, 500 mg/L). These media are marked “PVP” and “ac,” respectively. Some callus cultures were obtained from the corresponding suspension cell cultures according to the scheme “callus → suspension → callus.” A complete list of cell lines used and their media are given in Table 1.

Calli was grown in the dark at 26 °C. The fresh biomass gain (the callus-to-transplant weight ratio) of callus cell cultures was determined on the 56th day of growth, as described earlier [43].

#### 4.1.2. Suspension Cell Cultures 

Suspension cell cultures were obtained from the corresponding callus cultures using a standard procedure [43]. Suspension cultures of *T. × media*, *T. canadensis*, and *T. baccata* cells were obtained in 2009–2010, suspension cell culture of *T. wallichiana* was developed in 2016 [44].

Suspension cell cultures were grown in media containing Gamborg (B5) mineral salts. The medium composition for each suspension cell line was similar to the corresponding callus line except for the absence of agar. The suspension cell cultures were grown in the dark using an orbital shaker (100 ± 10 rpm) at 26 ± 1 ° C and relative humidity of 70 ± 5%. Flasks of 250, 500, and 1000 mL volume filled with, respectively, 40, 80, and 160 mL of culture medium were used for the cultivation of cell suspensions. Cultures were transferred to the fresh medium (subcultured) on the 28th day of cultivation; the inoculum-to-fresh medium ratio was 1:4. To characterize the growth and physiological state of the cell cultures, the dry and fresh weights of the cells and their viability were determined as described earlier [43,44].

The growth index (***I***), specific growth rate (**μ**), biomass doubling time (**τ**), economic coefficient (***Y***), and biomass productivity (***P***) were calculated using the following equations [45,46]:

***I***= *X*_max_/*X*_0_,

where *X*_max_ and *X*_0_ are the maximum and initial values of the growth criterion (dry or fresh weight of cells), respectively;

**µ** (day^–1^) = (ln *X*_2_ − ln *X*_1_)/(*t*_2_ − *t*_1_),

where *X*_2_ and *X*_1_ are the values of the growth criterion (dry or fresh weight of cells) at time points *t*_2_ and *t*_1_, respectively (calculated for the exponential growth phase);

**τ** (day) = ln 2/µ;

***Y***= (*X*_max_ − *X*_0_)/*S*_0_,

where *X*_max_ and *X*_0_ are the maximum and initial concentrations of dry cell biomass (g/L), respectively, and *S*_0_ is the initial concentration of the substrate (sucrose) in the medium (g/L of the medium);

***P***(g/L day) = (*X_i_* − *X*_0_)/(*t_i_* − *t*_0_),

where *X*_0_ and *X_i_* are the amounts of dry biomass at the beginning of cultivation and at time *t_i_*, respectively.

In addition, a suspension culture of *T. baccata* cells (**Tb-msu**/B5-NB) was grown in a 20-L bubble-type conical bioreactor designed at the Department of Cell Biology and Biotechnology, Timiryazev Institute of Plant Physiology, Russian Academy of Sciences, with a total volume of 20 L and a working volume of 15 L [47]. A cell culture grown in flasks was used as an inoculum; the density of the inoculum was 2 g/L by dry weight. Depending on the growth cycle phase, the air was supplied at a rate of 0.1–1.0 L/L/min. The concentration of dissolved oxygen pO_2_ was maintained at 10–40% of saturation in the absence of intense foaming.

### 4.2. Biochemical Analysis of the Cell Cultures

#### 4.2.1. Sample Preparation for Taxoids Screening

A sample (25 mg) of powdered air-dry biomass or dried culture medium was extracted three times with 1 mL of 96% ethanol for 30 min in an ultrasonic bath (Sapfir, Moscow, Russia), then centrifuged at 130,000 rpm for 10 min; the supernatant was collected into a pear-shaped flask. The combined alcohol extracts were evaporated under vacuum by heating to 50 °C in a water bath. The resulting dry extract was dissolved in 1 mL of distilled water and applied to a Supelclean ENVI-18 solid-phase extraction cartridge (Supelco, St. Louis, Missouri, USA). The cartridge was washed with 3 mL of water, and the analytes were desorbed with 3 mL of ethanol. The resulting solution was evaporated in a vacuum at 50 °C. Before analysis, the extracts were dissolved in an acetonitrile–water mixture (1:1, by volume). When analyzing the culture media of suspension cell cultures, 10 mL of the medium was applied to a cartridge for solid-phase extraction. Further preparation of samples was carried out according to the procedure described above. For most cell cultures, the analysis was carried out for two to four different subcultures (in the tables, the results for different subcultures are presented separately).

#### 4.2.2. UPLC–ESI-MS Analysis of Taxoids

The analysis was performed using a Waters Acquity UPLC system (Waters, Milford, MA, USA) equipped with a Xevo QTof hybrid quadrupole time-of-flight mass spectrometer (Waters, Milford, MA, USA). A sample (1 μL) was injected in an ACQUITY UPLC BEH Phenyl column (50 × 2.1 mm, 1.7 μm; Waters, Drinagh, County Wexford, Ireland). The column temperature was 40 °C, and the flow rate of the mobile phase was 0.4 mL/min. A 0.1% (by volume) solution of formic acid in water (solvent A) and a 0.1% (by volume) solution of formic acid in acetonitrile (solvent B) were used as the mobile phase.

Chromatographic separation was performed in the gradient elution mode. The gradient elution was performed by the following program (B, % by volume): 0–1 min, 35%; 1–7 min, 35 → 45%; 7–17 min, 45%; 17–17.5 min, 45 → 95%; 17.5–19 min, 95%; 19–19.5 min, 95 → 35%.

The analysis was carried out in the positive-ion mode in the *m*/*z* range of 100–1200. Ionization source parameters were as follows: ionization source temperature 120 °C; desolvation temperature 250 °C; capillary voltage 3.0 kV; sample injection cone voltage 30 V; nitrogen (desolvation gas) flow rate 600 L/h.

Commercial standard samples of baccatin III, cephalomannine, paclitaxel (Sigma-Aldrich, St. Louis, MO, USA), 10-deacetyl-7-xylosyl taxol, and taxusin (ChromaDex, Irvine, CA, USA) were used to identify 13-OH taxoids.

#### 4.2.3. Preparative Isolation of Taxoids from *T. baccata* Cell Culture

Preparative isolation of diterpenoids was performed using 93 g of air-dry biomass from a suspension culture of *T. baccata* cells (line **Tb-msu**/B5-NB) grown in a 20-L bubble-type bioreactor. Diterpenoids were separated using a combination of classical column chromatography with silica gel (Silicagel 60, grade 7734, 70-230 mesh, Sigma-Aldrich, St. Louis, MO, USA) and semi-preparative TLC (Uniplate Silica gel GF, Analtech, Newark, DE, USA) according to published procedures [28,29].

#### 4.2.4. High-Resolution Mass Spectrometry

High-resolution mass spectra with electrospray ionization were recorded using a Bruker micrOTOF II instrument as described earlier [48].

#### 4.2.5. NMR Spectroscopy

The ^1^H and ^13^C NMR spectra of the isolated compounds were measured in chloroform-d using a Bruker Avance AV600 instrument (Germany); tetramethylsilane was used as the internal standard. Signals in the ^1^H and ^13^C NMR spectra were assigned using two-dimensional NMR experiments (^1^H–^1^H COSY, TOCSY, ^1^H–^13^C HSQC, and HMBC).

## Figures and Tables

**Table 1:** Callus cell cultures of *Taxus* spp. with nutrient media used for their initiation and maintenance, and increase in fresh weight.

Species and Donor Plant Location	Cell Line */ Initiation Medium (Im)	Maintenance Medium	Fresh Weight Gain ± s.d.
***Taxus × media*** cv. **Dovastaniana** Botanical garden of Moscow State University	**TmD-msu**/W-DK	**Im** (W-DK) B5-NB R-PB-ac	3.1 ± 0.8 8.4 ± 0.1 2.6 ± 0.2
***Taxus × media*** cv. Aureovariegata Botanical garden of Moscow State University	**TmA-msu**/B5-NB	**Im** (B5-NB)	<1.1
		B5-NB-pvp	<1.1
	**TmA-msu**/R-PB-ac	**Im** (R-PB-ac)	3.4 ± 0.1
	**TmA-msu**/R-NB-ac	**Im** (R-NB-ac)	8.0 ± 0.1
	**TmA-msu**/R-DK-pvp	**Im** (R-DK-pvp)	6.5 ± 1.6
***Taxus baccata*** Botanical garden of Moscow State University	**Tb-msu**/B5-NB	**Im** (B5-NB) B5-NB-pvp **Im** (B5-NB)→susp→**Im**	<1.1 <1.1 4.9 ± 0.6
	**Tb-msu**/B5-PB	**Im** (B5-PB)	<1.1
		B5-PB-pvp	<1.1
	**Tb-msu**/B5-DK	**Im** (B5-DK)	<1.1
		B5-DK-pvp	<1.1
	**Tb-msu**/R-PB-ac	**Im** (R-PB-ac)	<1.1
		**Im** (R-PB-ac)→susp→**Im**	1.7 ± 0.4
	**Tb-msu**/R-NB-ac	**Im** (R-NB-ac)	<1.1
	**Tb-msu**/R-NB-pvp	**Im** (R-NB-pvp)	<1.1
***Taxus baccata*** Nikitsky botanical garden (Crimea)	**Tb-nbg**/B5-NB	**Im** (B5-NB)	3.3 ± 0.8
	**Tb-nbg**/B5-PB	**Im** (B5-PB)	<1.1
	**Tb-nbg**/B5-DK-ac	**Im** (B5-DK-ac)	8.2 ± 2.8
	**Tb-nbg**/B5-PB-ac	**Im** (B5-PB-ac)	1.6 ± 0.2
	**Tb-nbg**/R-PB-ac	**Im** (R-PB-ac)	3.7 ± 0.3
		B5-PB-ac	7.5 ± 0.5
		B5-PB	1.7 ± 0.8
	**Tb-nbg**/R-NB-ac	**Im** (R-NB-ac)	3.2 ± 0.4
		B5-PB	<1.1
		B5-PB-ac	9.1 ± 0.6
	**Tb-nbg**/R-DK-pvp	**Im** (R-DK-pvp)	1.2 ± 0.1
	**Tb-nbg**/R-NB-pvp	**Im** (R-NB-pvp)	3.6 ± 1.9
	**Tb-nbg**/R-DK-ac	**Im** (R-DK-ac)	4.5 ± 0.9
***Taxus canadensis*** Botanical garden of Moscow State University	**Tc-msu**/R-PB-pvp	**Im** (R-PB-pvp)	2.8 ± 0.3
		**Im** (R-PB-pvp)→susp→**Im**	1.5 ± 0.1
***Taxus wallichiana*** Botanical garden of Belarus Academy of Sciences	**Tw-bbg**/B5-NB-pvp	**Im** (B5-NB-pvp)	4.1 ± 0.2
		

* **Cell line abbreviations:** Species indication: TmD—*Taxus × media* cv. Dovastaniana; TmA—*Taxus × media* cv. Aureovariegata; Tb—*Taxus baccata*; Tc—*Taxus canadensis*; Tw—*Taxus wallichiana*. Explant origin: msu—botanical garden of Moscow State University; 30–40-year-old trees; nbg—Nikitsky botanical garden, 800-year-old tree of *Taxus baccata*; bbg—Main botanical garden of Belarus Academy of Sciences, 50-year-old tree of *Taxus wallichiana.* Mineral salt base: B5—Gamborg medium, R—Reinart’s medium, W—White medium. Plant growth regulators: N—NAA, 2 mg/L; D—2,4-D, 1 mg/L; P—picloram, 1 mg/L; B—6-BAP, 0.3 mg/L; K—kinetin, 0.3 mg/L. Additives: pvp—polyvinylpyrrolidone 4000, 1.0 g/L; ac—activated charcoal, 500 mg/L. Susp—suspension cells culture; Im—initial medium (medium for callus induction).

**Table 2:** Growth characteristics of the suspension cell cultures of *Taxus baccata* grown in 250-mL flasks and 20-L bioreactors.

Line	M_max_ (g/L)	I	μ_max_ (day^−1^)	T (day)	Y	P (g/L Day)
**Tb-msu**/B5-PB-*pv*p, flask	9.2	5.8	0.22	3.1	0.30	0.36
**Tb-msu**/B5-NB, flask	11.3	8.1	0.20	3.4	0.38	0.42
**Tb-msu**/B5-NB, bioreactor	13.0	7.2	0.20	3.4	0.43	0.55

M_max—_maximum dry biomass accumulation, g/L. I—growth index based on dry weight. μ_max_—maximum specific growth rate based on dry weight, day^−1^. T—doubling time, day. Y—economic coefficient. P—productivity, g/L day.

**Table 3:** Results of UPLC–ESI-MS analysis (positive ion mode) of an alcohol extract from the biomass of *T. baccata* callus culture (**Tb-nbg** R-NB-*ac* line grown in a B5-PB-ac medium, 74 days of 42nd subculturing cycle). Peak numbers correspond to those in Figure 2.

Peak No.	t_R_, Min	[M + NH_4_]^+^, *m*/*z*		[M + Na]^+^, *m*/*z*		[M + K]^+^, *m*/*z*		Fragment Ions, m/z	Elemental Composition	Identification
		Exp. *	Calc. *	Exp.	Calc.	Exp.	Calc.			
1	3.7	538. 3062	538. 3016	543. 2529	543. 2570	559. 2319	559. 2309	461.2519; 401.2310; 359.2278; 341.2111; 299.1985; 281.1903; 263.1849	C_28_H_40_O_9_	7-Hydroxy-2,5,10,14-tetra- acetoxy-taxadiene
2	5.0	580. 3130	580. 3122	585. 2744	585. 2676	601. 2397	601. 2415	503.2673; 461.2498; 443.2375; 401.2319; 383.2209; 359.2213; 341.2102; 323.2006; 299.2037; 281.1903; 263.1767; 253.1957	C_30_H_42_O_10_	2,5,9,10,14-Penta-acetoxy- taxadiene
3	5.3	580. 3447	580. 3486	585. 2991	585. 3040	601. 2791	601. 2779	503.2989; 461.2935; 401.2885; 385.2409; 343.2278; 325.2160; 283.2061; 265.1956	C_31_H_46_O_9_	Yunnanxane
4	5.9	522. 3033	522. 3067	527. 2594	527. 2621	543. 2376	543. 2360	445.2578; 403.2489; 385.2387; 343.2321; 325.2183; 283.2072; 265.1946	C_28_H_40_O_8_	Taxuyunnanine C
5	9.0	622. 3594	622.3591	627. 3124	627. 3145	643. 2960	643. 2885	503.2976; 443.2478; 401.2302; 383.2262; 341.2102; 323.1978; 281.1893; 263.1836; 253.1904	C_33_H_48_O_10_	Taxuyunnanine B
6	10.7	564. 3470	564. 3536	569. 3086	569. 3090	585. 2853	585. 2830	487.4061; 469.2935; 445.2933; 427.2852; 325.2157; 283.2042; 265.1919; 255.2110	C_31_H_46_O_8_	Sinenxane C

* Exp.—experimental value; * Calc.—calculated value.

**Table 4:** Results of UPLC–ESI-MS analysis (positive ion mode) of an alcohol extract from the biomass of *T. baccata* cell suspension culture (**Tb-msu**/B5-NB-ac line grown in a 20-L bioreactor). Peak numbers correspond to those in Figure 3.

Peak No.	t_R_, Min	[M + NH_4_]^+^, *m*/*z*		[M + Na]^+^, *m*/*z*		[M + K]^+^, *m*/*z*		Fragment Ions, m/z	Elemental Composition	Identification
		Exp. *	Calc. *	Exp.	Calc.	Exp.	Calc.			
1	3.7	538. 2882	538. 3016	543. 2548	543. 2570	559. 2460	559. 2309	461.2505; 401.2343; 299.1857; 281.1884	C_28_H_40_O_9_	7-Hydroxy-2,5,10,14-tetra- acetoxy-taxadiene
2	5.0	580. 3076	580. 3122	585. 2753	585. 2676	601.2474	601. 2415	461.2549; 443.2408; 401.2318; 383.2212; 359.2234; 341.2069; 323.1993; 299.1989; 281.1774; 263.1734	C_30_H_42_O_10_	2,5,9,10,14-Penta-acetoxy- taxadiene
3	5.3	580. 3441	580. 3486	585. 2919	585. 3040	601. 2843	601. 2779	503.3022; 461.2924; 385.2391; 343.2255; 325.2200; 283.2014; 265.1931	C_31_H_46_O_9_	Yunnanxane
4	5.9	522. 2980	522. 3067	527. 2594	527. 2606	543. 2376	543. 2335	445.2589; 403.2479; 385.2302; 343.2222; 325.2097; 283.1939; 265.1988	C_28_H_40_O_8_	Taxuyunnanine C
5	7,4	536. 3209	536. 3223	541. 2850	541. 2702	557. 2466	557. 2517	441.2720; 417.2695; 385.2421; 343.2316; 325.2180; 283.2086; 265.1936	C_29_H_42_O_8_	Sinenxane B
6	8.9	550. 3374	550. 3380	555. 2829	555. 2934	571. 2705	571. 2632	445.2998; 431.2837; 385.2393; 343.2289; 325.2186; 283.2039; 265.1943	C_30_H_44_O_8_	2,5,10-Triacetoxy-14-(iso-butyryloxy)-taxadiene
7	9.0	622. 3586	622. 3591	627. 3124	627. 3128	643. 2960	643. 2954	503.2985; 443.2540; 401.2372; 383.2262; 341.2217; 323.2025; 281.1904; 263.1782; 253.1917	C_33_H_48_O_10_	Taxuyunnanine B
8	10.6	564. 3470	564. 3407	569. 3086	569. 3076	585. 2853	585. 2827	487.3022; 469.2929; 445.2925; 427.2793; 385.2319; 343.2233; 325.2115; 283.1945; 265.1841; 255.2095	C_31_H_46_O_8_	Sinenxane C

* Exp.—experimental value; * Calc.—calculated value.

**Table 5:** Taxoids identified in callus cell cultures of *Taxus* spp.

Species/ Donor Tree Location	Cell Line/Initiation Medium (Im)	Variant or Growth Medium	No. of Subcultivation	Days of Subcultivation	Detected Taxoids *							
					1	2	3	4	5	6	7	8
*T. × media* cv. Dovastaniana/ MSU botanical garden	TmD-msu /W-DK	**Im** (W-DK)	53	74								
		B5-NB	51	126								
			52	91								
		R-PB-*ac*	47	74								
*T. × media* cv. Aureovariegata/ MSU botanical garden	TmA-msu /B5-NB	**Im** (B5-NB)	42	57								
			45	126								
	TmA-msu /R-PB-*ac*	**Im** (R-PB-*ac*)	42	73								
			47	74								
	TmA-msu /R-NB-*ac*	**Im** (R-NB-*ac*)	42	73								
			47	74								
	TmA-msu/R- DK-*pvp*	**Im** (R-DK- *pvp*)	43	37								
			47	70								
*T. baccata*/ MSU botanical garden	Tb-msu/B5-NB	**Im**→susp→ →**Im** (B5-NB)	8 (41)	50								
			10 (43)	34								
			10 (43)	126								
	Tb-msu /R-PB-*ac*	**Im** (R-PB-*ac*)	37	50								
		**Im**→susp→ →**Im** (R-PB-ac)	8 (41)	43								
	Tb-msu/R-NB-*ac*	**Im** (R-NB-ac)	44	31								
	Tb-msu/R-NB-*pvp*	**Im** (R-NB-pvp)	42	70								
*T. baccata*/ Nikitski botanical garden (Crimea)	**Tb-nbg**/B5-NB	**Im** (B5-NB)	36	70								
			37	24								
			39	126								
			40	91								
	**Tb-nbg**/B5-PB	**Im** (B5-PB)	41	96								
	**Tb-nbg**/B5-DK-*ac*	**Im** (B5-DK-*ac*)	36	73								
			44	80								
	**Tb-nbg**/B5-PB-*ac*	**Im** (B5-PB-*ac*)	37	70								
			38	37								
			44	80								
	**Tb-nbg**/R-PB-*ac*	**Im** (R-PB-*ac*)	37	24								
			37	70								
			40	136								
			41	61								
		B5-PB-*ac*	42	74								
	**Tb-nbg**/R-NB-*ac*	**Im** (R-NB-*ac*)	44	80								
		B5-PB-*ac*	36	70								
			42	74								
	**Tb-nbg**/R-DK- *pvp*	**Im** (R-DK- *pvp*)	42	70								
	**Tb-nbg**/R-DK-*ac*	**Im** (R-DK-*ac*)	38	37								
			44	80								
*Taxus canadensis*/ MSU botanical garden	**Tc-msu**/R-PB- *pvp*	**Im** (R-PB-*pvp*)	42	70								
		**Im**→susp→ →**Im** (R-PB-*pvp)*	10 (43)	70								
			13 (46)	136								
			14 (47)	70								
*Taxus Wallichiana*	**Tb-bbg**/B5-NB-*pvp*	**Im** (B5-NB-*pvp*)	5	91								

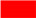
—taxoid is detected in cell biomass; *—1–8—detected compounds (taxoids) according to Table 4: **1**—7-hydroxy-2,5,10,14-tetra-acetoxytaxadiene; **2**—2,5,9,10,14-penta-acetoxytaxadiene; **3**—Yunnanxane; **4**—Taxuyunnanine C; **5**—Sinenxane B; **6**—2,5,10-tri-acetoxy-14-(iso-butyryloxy)-taxadiene; **7**—Taxuyunnanine B; **8** — Sinenxane C.

**Table 6:** Taxoids identified in suspension cell cultures of *Taxus* spp.

Species/Cell Line	Growth Medium	No. of Subcultivation	Days of Subcultivation	Detected Taxoids *							
				1	2	3	4	5	6	7	8
T. × media cv. Aureovariegata/TmA-msu/B5-NB	**Im** (B5-NB)	70	27								
		78	27								
		90	33								
	B5-NB-*pvp*	78	27								
		90	33								
*T. baccata*/ **Tb-msu**/B5-NB	**Im** (B5-NB)	70	21								
		83	27								
		84	21								
		91	21								
	B5-NB-*pvp*	91	21								
*T. baccata*/ **Tb-msu**/B5-PB	**Im** (B5-PB)	81	27								
		85	21								
	B5-PB-*pvp*	81	27								
		90	33								
*T. baccata/* **Tb-msu**/B5-DK	**Im** (B5-DK)	83	27								
		91	27								
	B5-DK-*pvp*	83	27								
		91	21								
*T.wallichiana*/ **Tw-nbb**/B5-NB-*pvp*	**Im** (B5-NB-*pvp)*	21	4								

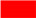
—taxoid is detected in cell biomass; *—**1**–**8**—detected compounds (taxoids) according to Table 4: **1**—7-hydroxy-2,5,10,14-tetra-acetoxytaxadiene; **2**—2,5,9,10,14-penta-acetoxytaxadiene; **3**—Yunnanxane; **4**—Taxuyunnanine C; **5**—Sinenxane B; **6**—2,5,10-tri-acetoxy-14-(iso-butyryloxy)-taxadiene; **7**—Taxuyunnanine B; **8**—Sinenxane C.

## Data Availability

All data used in this study was gathered from open literature sources or scientific journals under institutional subscription.

## Supplementary Materials

The following supporting information can be downloaded at: https://www.mdpi.com/article/10.3390/molecules28052178/s1, Table S1: ^13^C NMR spectra (125 MHz, CDCl_3_) of taxoids I–V isolated from *T. baccata* cell suspension culture; Table S2: ^1^H NMR spectra (600 MHz, CDCl_3_) of taxoids I–V isolated from *T. baccata* cell suspension culture; Table S3: Taxoids detected in culture medium of the callus cell lines of *Taxus* spp.; Table S4: Taxoids detected in the cultivation medium of suspension cell cultures of *Taxus* spp.
