# Supplementary material for: Profiling of Taxoid Compounds in Plant Cell Cultures of Different Species of Yew (Taxus spp.)
Source: Molecules. 2023 Feb 26;28(5):2178. doi: 10.3390/molecules28052178 (PMC10004465; doi:10.3390/molecules28052178)
Supplement: Supplementary file 1 [file molecules-28-02178-s001.zip › molecules-2158854-supplementary.pdf]

Supplementary Materials:

Table S1. ^{13}C NMR spectra (125 MHz, $CDCl_3$) of taxoids **I–V** isolated from *T. baccata* cell suspension culture

Position in a molecule/ Taxadiene skeleton	Taxoid				
	V	IV	III	II	I
1	59.31	59.32	58.60	59.5	59.3
2	70.61	70.59	70.28	70.76	70.01
3	42.19	42.15	44.22	42.3	40.05
4	142.32	142.33	141.51	141.9	140.09
5	78.20	78.28	78.37	78.24	77.7
6	28.92	28.89	28.37	29.69	37.0
7	33.86	33.84	28.37	33.85	69.01
8	39.9	39.49	44.44	39.52	44.4
9	43.94	43.92	77.00	44.2	37.0
10	70.15	70.13	72.52	70.08	69.7
11	135.39	135.42	132.88	135.5	136.01
12	134.85	134.75	137.30	134.5	134.09
13	39.69	39.49	39.51	39.52	39.6
14	70.15	70.59	69.94	70.76	70.05
15	37.9	37.34	37.07	38.78	37.7
16	25.43	25.44	25.90	25.40	25.3
17	31.72	31.77	31.66	30.39	32.0
18	20.94	20.93	21.38	20.86	21.5
19	22.97	22.47	17.36	22.49	16.6
20	116.82	116.94	118.15	117	118.1
**Acyl substituents**					
$CH_3CO \rightarrow C2$					
CO	169.80	169.95	169.75/169.94	169.9	170.0
CH_3	21.35	21.41	21.04/20.75	21.39	21.3
$CH_3CO \rightarrow C5$					
CO	169.95	170.05	169.75/169.94	170.2	170.0
CH_3	21.41	21.41	21.04/20.75	22.49	21.3
$CH_3CO \rightarrow C9$					
CO	–	–	170.04	–	–
CH_3	–	–	21.04/20.75	–	–
$CH_3CO \rightarrow C10$					
CO	170.23	169.77	169.75/169.94	169.9	169.5
CH_3	21.89	21.82	21.83	21.39	22.3
acyl→C14					
C1′ (CO)	175.63	–	–	174.9	–
C2′	41.11	–	–	47.4	–
C3′	26.75	–	–	69.52	–
C4′ (CH_3)	11.59	–	–	20.86	–
C5′ (CH_3)	16.57	–	–	14.02	–
$CH_3CO \rightarrow C14$					
CO	–	170.24	169.75/169.94	–	171.2
CH_3	–	21.41	21.04/20.75	–	21.8

Table S2. ^{1}H NMR spectra (600 MHz, $CDCl_3$) of taxoids **I–V** isolated from *T. baccata* cell suspension culture.

Position in a molecule	Taxoid V	IV	III	II	I
1	1H, 1.883, *d*, *J* = 2.4 Hz, H-1	1H, 1.899, *d*, *J* = 2.4 Hz, H-1	1H, 1.955, *d*, *J* = 2.4 Hz, H-1	1H, 1.914, *d*, *J* = 1.8 Hz, H-1	1H, 1.929, *d*, *J* = 1.8 Hz, H-1
2	1H, 5.365, *dd*, *J* = 6.6, 2.4 Hz, H-2	1H, 5.363, *dd*, *J* = 6.0, 2.4 Hz, H-2	1H, 5.431, *dd*, *J* = 6.6, 1.8 Hz, H-2	1H, 5.374, *dd*, *J* = 6.6, 2.4 Hz, H-2	1H, 5.417, *dd*, *J* = 6, 2.4 Hz, H-2
3	1H, 2.951, *d*, *J* = 6.6 Hz, H-3	1H, 2.938, *d*, *J* = 6.0 Hz, H-3	1H, 2.986, *d*, *J* = 6.6 Hz, H-3	1H, 2.943, *d*, *J* = 6.6 Hz, H-3	1H, 2.779, *d*, *J* = 6.6 Hz, H-3
5	1H, 5.302, *t*, *J* = 3 Hz, H-5	1H, 5.300, *t*, *J* = 3 Hz, H-5	1H, 5.323, *t*, *J* = 3 Hz, H-5	1H, 5.309, *t*, *J* = 3 Hz, H-5	1H, 5.352, *t*, *J* = 3.6 Hz, H-5
6	1H, 1.726, *m*, H-6	1H, 1.816, *m*, H-6	1H, 1.852, *m*, H-6	1H, 1.821, *m*, H-6	1H, 2.131, *m*, H-6α
6	–	–	–	–	1H, 1.649, *m*, H-6β
7α	1H, 1.975, *dt*, *J* = 13.2, 7.2 Hz, H-7α	1H, 1.977, *dt*, *J* = 13.2, 7.2 Hz, H-7α	1H, 1.694, *m*, H-7β+α	1H, 1.974, *m*, H-7α	1H, 3.915, *dd*, *J* = 12, 5.1 Hz, H-7α
7β	1H, 1.262, *m*, H-7β	1H, 1.264, *m*, H-7β	–	1H, 1.266, *m*, H-7β	–
9α	1H, 1.637, *m*, H-9α	1H, 1.642, *dd*, *J* = 15, 5.4 Hz, H-9α	1H, 5.817, *d*, *J* = 10.2 Hz, H-9	1H, 1.651, *dd*, *J* = 14.4, 5.4 Hz, H-9α	1H, 2.131, *m*, H-9α
9β	1H, 2.396, *m*, H-9β	1H, 2.391, *m*, H-9β	–	1H, 2.397, *m*, H-9β	1H, 2.230, *dd*, *J* = 15, 6 Hz, H-9β
10	1H, 6.069, *dd*, *J* = 12, 5.4 Hz, H-10	1H, 6.070, *dd*, *J* = 12, 5.4 Hz, H-10	1H, 6.031, *d*, *J* = 10.8 Hz, H-10	1H, 6.072, *dd*, *J* = 12, 5.4 Hz, H-10	1H, 5.988, *dd*, *J* = 12, 4.8 Hz, H-10
13α	1H, 2.862, *dd*, *J* = 18.9, 9.3 Hz, H-13α	1H, 2.824, *dd*, *J* = 18.9, 9.3 Hz, H-13α	1H, 2.858, *dd*, *J* = 19.2, 9.6 Hz, H-13α	1H, 2.862, *dd*, *J* = 19.2, 9.6 Hz, H-13α	1H, 2.804, *dd*, *J* = 18.6, 9.0 Hz, H-13α
13β	1H, 2.384, *dd*, *J* = 18.6, 4.7 Hz, H-13β	1H, 2.426, *dd*, *J* = 18.6, 4.8 Hz, H-13β	1H, 2.453, *dd*, *J* = 19.2, 4.8 Hz, H-13β	1H, 2.403, *m*, H-13β	1H, 2.434, *dd*, *J* = 19.8, 4.8 Hz, H-13β
14	1H, 4.998, *dd*, *J* = 9, 4.8 Hz, H-14	1H, 5.006, *dd*, *J* = 9, 4.8 Hz, H-14	1H, 4.989, *dd*, *J* = 9.6, 4.8 Hz, H-14	1H, 5.048, *dd*, *J* = 9.6, 4.8 Hz, H-14	1H, 4.974, *dd*, *J* = 9, 4.2 Hz, H-14
16	3H, 1.674, *s*, Me-16	3H, 1.669, *s*, Me-16	3H, 1.728, *s*, Me-16	3H, 1.679, *s*, Me-16	3H, 1.699, *s*, Me-16
17	3H, 1.132, *s*, Me-17	3H, 1.132, *s*, Me-17	3H, 1.139, *s*, Me-17	3H, 1.139, *s*, Me-17	3H, 1.157, *s*, Me-17
18	3H, 2.097, *s*, Me-18	3H, 2.098, *s*, Me-18	3H, 2.156, *s*, Me-18	3H, 2.106, *s*, Me-18	3H, 2.080, *s*, Me-18
19	3H, 0.852, *s*, Me-19	3H, 0.851, *s*, Me-19	3H, 0.866, *s*, Me-19	3H, 0.859, *s*, Me-19	3H, 0.765, *s*, Me-19
20α	1H, 5.268, *s*, H-20α	1H, 5.276, *s*, H-20α	1H, 5.346, *s*, H-20α	1H, 5.285, *s*, H-20α	1H, 5.306, *s*, H-20α
20β	1H, 4.826, *s*, H-20β	1H, 4.868, *t*, *J* = 1.2, H-20β	1H, 4.894, *s*, H-20β	1H, 4.841, *s*, H-20β	1H, 4.955, *s*, H-20β

Position in a molecule	Taxoid				
	V	IV	III	II	I
2′	1H, 2.321, *m*, H-2′	–	–	1H, 2.397, *m*, H-2′	–
3′	1H, 1.451, *m*, H-3′α	–	–	1H, 3.870, *m*, H-3′	–
3′	1H, 1.637, *m*, H-3′β	–	–	–	–
4′	3H, 0.892, *t*, *J* = 7.2 Hz, H-4′	–	–	3H, 1.218, *d*, *J* = 7.2 Hz, H-4′	–
5′	3H, 1.119, *d*, *J* = 7.2 Hz, H-5′	–	–	3H, 1.117, *d*, *J* = 7.2 Hz, H-5′	–
2-OAc	3H, 2.021, *s*, Ac2-Me	3H, 2.021, *s*, Ac2-Me	3H, 2.023, *s*, Ac2-Me	3H, 2.034, *s*, Ac2-Me	3H, 2.050, *s*, Ac2-Me
5-OAc	3H, 2.061, *s*, Ac5-Me	3H, 2.056, *s*, Ac5-Me	3H, 2.050, *s*, Ac5-Me	3H, 2.067, *s*, Ac5-Me	3H, 2.028, *s*, Ac5-Me
10-OAc	3H, 2.097, *s*, Ac10-Me	3H, 2.178, *s*, Ac10-Me	3H, 2.194, *s*, Ac10-Me	3H, 2.192, *s*, Ac10-Me	3H, 2.182, *s*, Ac10-Me
14-OAc	–	3H, 2.062, *s*, Ac14-Me	3H, 2.055, *s*, Ac14-Me	–	3H, 2.067, *s*, Ac14-Me
9-OAc	–	–	3H, 2.023, *s*, Ac9-Me	–	–
6	1H, 1.883, *d*, *J* = 2.4 Hz, H-1	1H, 1.899, *d*, *J* = 2.4 Hz, H-1	1H, 1.955, *d*, *J* = 2.4 Hz, H-1	1H, 1.914, *d*, *J* = 1.8 Hz, H-1	1H, 1.929, *d*, *J* = 1.8 Hz, H-1
7α	1H, 5.365, *dd*, *J* = 6.6, 2.4 Hz, H-2	1H, 5.363, *dd*, *J* = 6.0, 2.4 Hz, H-2	1H, 5.431, *dd*, *J* = 6.6, 1.8 Hz, H-2	1H, 5.374, *dd*, *J* = 6.6, 2.4 Hz, H-2	1H, 5.417, *dd*, *J* = 6, 2.4 Hz, H-2
7β	1H, 2.951, *d*, *J* = 6.6 Hz, H-3	1H, 2.938, *d*, *J* = 6.0 Hz, H-3	1H, 2.986, *d*, *J* = 6.6 Hz, H-3	1H, 2.943, *d*, *J* = 6.6 Hz, H-3	1H, 2.779, *d*, *J* = 6.6 Hz, H-3
9α	1H, 5.302, *t*, *J* = 3 Hz, H-5	1H, 5.300, *t*, *J* = 3 Hz, H-5	1H, 5.323, *t*, *J* = 3 Hz, H-5	1H, 5.309, *t*, *J* = 3 Hz, H-5	1H, 5.352, *t*, *J* = 3.6 Hz, H-5
9β	1H, 1.726, *m*, H-6	1H, 1.816, *m*, H-6	1H, 1.852, *m*, H-6	1H, 1.821, *m*, H-6	1H, 2.131, *m*, H-6α
10	–	–	–	–	1H, 1.649, *m*, H-6β
13α	1H, 1.975, *dt*, *J* = 13.2, 7.2 Hz, H-7α	1H, 1.977, *dt*, *J* = 13.2, 7.2 Hz, H-7α	1H, 1.694, *m*, H-7β+α	1H, 1.974, *m*, H-7α	1H, 3.915, *dd*, *J* = 12, 5.1 Hz, H-7α
13β	1H, 1.262, *m*, H-7β	1H, 1.264, *m*, H-7β	–	1H, 1.266, *m*, H-7β	–

Table S3.Taxoids detected in culture medium of the callus cell lines of *Taxus spp.*

Species / donor tree location	Cell line /Initiation medium (Im)	Variant or growth medium	No. of sub-cultivation	Days of sub-cultivation	Detected taxoids* 1	2	3	4	5	6	7	8
T. × media cv. Dovastaniana/ MSU botanical garden	TmD-msu /W-DK	**Im** (W-DK)	53	74								
		B5-NB	51	126		■	■					■
			52	91								
		R-PB-*ac*	47	74								
T. × medi cv. Auredvaregate/ MSU botanical garden	TmA-msu /B5-NB	**Im** (B5-NB)	45	126								
	TmA-msu /R-PB-*ac*	**Im** (R-PB-*ac*)	47	74	■	■	■	■			■	■
	TmA-msu /R-NB-*ac*	**Im** (R-NB-*ac*)	47	74	■	■	■	■			■	■
	TmA-msu/R- DK-*pvp*	**Im** (R-DK- *pvp*)	47	70	■	■	■	■			■	■
T. baccata/ MSU botanical garden	Tb-msu/B5-NB	**Im** → susp→ →**Im** (B5-NB)	10 (43)	34	■	■	■					■
			10 (43)	126	■	■	■					■
		**Im** → susp→ → **Im** (R-PB-ac)	8 (41)	43								
T. baccata/ Nikitski botanical garden (Crimea)	**Tb-nbg**/B5-NB	**Im** (B5-NB)	39	126	■	■	■					■
			40	91		■	■	■				■
	**Tb-nbg**/B5-DK-*ac*	**Im** (B5-DK-*ac*)	44	80	■	■	■	■	■		■	■
	**Tb-nbg**/B5-PB-*ac*	**Im** (B5-PB-*ac*)	38	37								
			44	80		■	■	■			■	■
	**Tb-nbg**/R-PB-*ac*	**Im** (R-PB-*ac*)	40	136	■	■	■	■				■
			41	61	■	■	■	■			■	■
		B5-PB-*ac*	42	74	■	■	■	■				■
		B5-PB	41	96	■	■	■				■	■
	**Tb-nbg**/R-NB-*ac*	**Im** (R-NB-*ac*)	44	80	■	■	■				■	■
		B5-PB-*ac*	42	74								
	**Tb-nbg**/R-DK- *pvp*	**Im** (R-DK- *pvp*)	42	70					■			
	**Tb-nbg**/R-NB-pvp	**Im** (R-NB-*pvp*)	42	70			■	■				■
	**Tb-nbg**/R-DK-*ac*	**Im** (R-DK-*ac*)	44	80	■	■	■				■	■
Taxus canadensis/ MSU botanical garden	**Tc-msu**/R-PB- *pvp*	**Im** → susp→ →**Im** (R-PB-*pvp*)	13 (46)	136	■	■	■	■	■			■
			14 (47)	70	■	■	■	■	■			■
Taxus Wallichiana	**Tb-bbg**/B5-NB-*pvp*	**Im** (B5-NB-*pvp*)	5	91			■	■				■

■ taxoid is detected in cell biomass; **1 – 8** - detected compounds (taxoids) according to Table 4:

1 - 7-hydroxy-2,5,10,14-tetra-acetoxytaxadiene; **2** - 2,5,9,10,14-penta-acetoxytaxadiene;
3 – Yunnanxane; **4** - Taxuyunnanine C; **5** - Sinenxane B;
6 - 2,5,10-tri-acetoxy-14-(iso-butyryloxy)-taxadiene; **7** - Taxuyunnanine B; **8** - Sinenxane C;

Table S4 Taxoids detected in the cultivation medium of suspension cell cultures of *Taxus spp.*

Species / Cell line	Growth medium	No. of sub-cultivation	Days of sub-cultivation	Detected taxoids*							
				**1**	**2**	**3**	**4**	**5**	**6**	**7**	**8**
T. × media cv. Auredvaregate /TmA-msu/B5-NB	**Im** (B5-NB	70	27								
	B5-NB-*pvp*	78	27								
T. baccata / **Tb-msu/**B5-NB	**Im** (B5-NB)	84	21				■				■
	B5-NB-*pvp*	91	21								■
T. baccata / **Tb-msu/**B5-PB	**Im** (B5-PB)	91	27	■	■	■	■	■	■		■
T. baccata / **Tb-msu/**B5-DK	**Im** (B5-DK)	83	27	■	■	■				■	■
	B5-DK-*pvp*	91	21	■	■	■					■
T.wallichiana/ **Tw-nbb**/B5-NB-*pvp*	**Im** (B5-NB-*pvp)*	21	4		■	■	■	■	■		■

■ taxoid is detected in cell biomass; **1 – 8** - detected compounds (taxoids) according to Table 4:

1 - 7-hydroxy-2,5,10,14-tetra-acetoxytaxadiene; **2** - 2,5,9,10,14-penta-acetoxytaxadiene;
3 – Yunnanxane; **4** - Taxuyunnanine C; **5** - Sinenxane B;
6 - 2,5,10-tri-acetoxy-14-(iso-butyryloxy)-taxadiene; **7** - Taxuyunnanine B; **8** - Sinenxane C;
